# 2-Deoxyglucose Suppresses ERK Phosphorylation in LKB1 and Ras Wild-Type Non-Small Cell Lung Cancer Cells

**DOI:** 10.1371/journal.pone.0168793

**Published:** 2016-12-29

**Authors:** Linlin Sun, Xiuju Liu, Haian Fu, Wei Zhou, Diansheng Zhong

**Affiliations:** 1 Tianjin Key Laboratory of Lung Cancer Metastasis and Tumor Microenvironment, Lung Cancer Institute, Tianjin Medical University General Hospital, Tianjin, P.R. China; 2 Department of Hematology and Medical Oncology, Emory University School of Medicine, Atlanta, Georgia, United States of America; 3 Department of Pharmacology and Emory Chemical Biology Discovery Center, Emory University School of Medicine, Atlanta, Georgia, United States of America; 4 Department of Pathology and Laboratory Medicine and the Department of Human Genetics Emory University School of Medicine, Atlanta, Georgia, United States of America; 5 Department of Medical Oncology, Tianjin Medical University General Hospital, Tianjin, P.R. China; H. Lee Moffitt Cancer Center & Research Institute, UNITED STATES

## Abstract

Tumor cells rely on aerobic glycolysis to generate ATP, namely the "Warburg" effect. 2-deoxyglucose (2-DG) is well characterized as a glycolytic inhibitor, but its effect on cellular signaling pathways has not been fully elucidated. Herein, we sought to investigate the effect of 2-DG on ERK function in lung cancer cells. We found that 2-DG inhibits ERK phosphorylation in a time and dose-dependent manner in lung cancer cells. This inhibition requires functional LKB1. LKB1 knockdown in LKB1 wildtype cells correlated with an increase in the basal level of p-ERK. Restoration of LKB1 in LKB1-null cells significantly inhibits ERK activation. Blocking AMPK function with AMPK inhibitor, AMPK siRNA or DN-AMPK diminishes the inhibitory effect of 2-DG on ERK, suggesting that 2-DG—induced ERK inhibition is mediated by LKB1/AMPK signaling. Moreover, IGF1-induced ERK phosphorylation is significantly decreased by 2-DG. Conversely, a subset of oncogenic mutants of K-Ras, the main upstream regulator of ERK, blocks 2-DG—induced LKB1/AMPK signaling. These findings reveal the potential cross-talk between LKB1/AMPK and ERK signaling and help to better understand the mechanism of action of 2-DG.

## Introduction

One of the primary hallmarks of cancer [1] is altered glucose metabolism. Tumor cells are known to ferment glucose to lactate in the presence of oxygen, i.e. “aerobic glycolysis” [2]. This process, known as the “Warburg Effect”, is proposed to benefit the growth and survival of cancer cells through several candidate mechanisms [3], including rapid production of ATP [4], promoting biosynthesis [5] and acidification of the tumor microenvironment [6], etc. Based on these mechanistic rationales, targeting glycolysis has been explored as a therapeutic approach for cancer treatment.

Of all the glycolysis inhibitors that have been evaluated, 2-deoxyglucose (2-DG) has been best characterized in animal models [7] and human clinical trials [8,9]. The glucose analogue 2-DG is converted by hexokinase to 2-DG-P [10], which cannot be further metabolized but is trapped inside the cell and allosterically inhibits hexokinase, the rate-limiting enzyme in glycolysis. By blocking glycolysis, 2-DG interferes with various biological processes. First, it induces energy stress by depleting intracellular ATP [11,12]. Second, it affects anabolic processes by decreasing the production of glycolytic intermediates which are the precursors of nucleotides, lipids or proteins [13]. Finally, it results in NADPH deficiency and disrupts the antioxidant defenses of cancer cells. Independent of glycolysis inhibition, 2-DG is also known to interfere with the N-linked glycosylation process because of its structural similarity to mannose [14]. 2-DG has been shown to exert indirect effects on various signaling pathways. For example, 2-DG represses the activity of mammalian target of rapamycin (mTOR) by activating LKB1/AMP-activated protein kinase (AMPK) signaling, an energetic stress-sensing signaling pathway [15]. In addition, we previously demonstrated that 2-DG treatment induced the activation of IGF-1 receptor (IGF1R) signaling [16,17].

2-DG can efficiently inhibit cell growth and invasion, and potently facilitate apoptosis in various cancer cells [14,18,19]. However, the underlying molecular mechanisms are not yet well understood. A catabolic block does not sufficiently explain the anti-tumor activity of 2-DG [20]. Extracellular signal-regulated kinase (ERK) cascades are key signaling pathways involved in the regulation of cancer cell proliferation, survival and invasion [21]. ERK1/2 is a downstream component of an evolutionarily conserved RAF/MEK/ERK signaling module that is activated by the Ras small GTPase. Ras is the second most frequently mutated gene in non-small cell lung cancer (NSCLC), with up to 30% of tumors containing K-Ras activating mutation [22]. Mutations in the Ras protein, primarily at residues G12, G13 or Q61, can inhibit the hydrolysis of GTP, rendering the proteins constitutively GTP-bound and activated [23]. In this study, we sought to investigate the effect of the glycolysis inhibitor 2-DG on ERK activation. We found that 2-DG inhibits ERK phosphorylation in a subset of NSCLC cells with wild-type LKB1 and K-Ras. Our findings uncover the potential cross-talk between LKB1/AMPK and ERK signaling and offer novel insights into the mechanism of action of 2-DG.

## Materials and Methods

### Reagents

Mouse monoclonal antibody against LKB1 (#ab15095) was purchased from Abcam, UK. Antibodies against total AMPK (#2532), p-AMPKα Thr172 (#2535), p-ACC (phospho-acetyl-CoA carboxylase) Ser79 (#3661), total ERK1/2 (#9102), p-ERK1/2 Thr202/Tyr204 (#9101), total AKT (#9272), p-AKT Thr473 (#9271), p-S6K Thr389 (#9105) and Kras (#3965) were purchased from Cell Signaling Technology, USA. Rabbit polyclonal anti-actin antibody was purchased from Sigma-Aldrich, USA. Mouse anti-Ras antibody was purchased from Millipore, Germany. 2-DG, puromycin and IGF-1 were purchased from Sigma—Aldrich, USA. LY294002 (a PI3K inhibitor) was purchased from LC Laboratories. Compound C (an inhibitor of AMPK) solution was purchased from Calbiochem, USA. The lentiviral LKB1 short hairpin RNA (shRNA) construct and a negative control construct that was created in the same vector system (pLKO.1) were purchased from Open Biosystems, USA. LKB1 shRNA ID# is TRCN408, and its target sequence is GCCAACGTGAAGAAGGAAATT. Lentiviral helper plasmids (pCMV-dR8.2 dvpr and pCMV-VSV-G) were obtained from Addgene, USA. Plasmids encoding wild-type (WT) LKB1 [24], dominant negative AMPK (DN-AMPK) [25] and EGFP-K-Ras mutants [26] were constructed as previously described.

### Cell culture

The LKB1 mutant (H460, A549, H157 and H23) and LKB1 WT (H226, H522, HOP92, H596, H1703, H520, Calu-1, H1650, HCC827 and H1975) NSCLC cell lines were purchased from the American Type Culture Collection (ATCC, USA). The isogenic K-Ras G13D and K-Ras WT HCT116 colon carcinoma cells were kindly obtained from Prof. Bert Vogelstein (The Johns Hopkins University School of Medicine, Baltimore, MD 21287, USA). Cells were cultured in RPMI 1640 medium (Gibco, USA) supplemented with 10% fetal bovine serum (Gibco, USA) at 37°C in a humidified atmosphere with 5% CO_2_.

### LKB1 stable knockdown using lentiviral short hairpin RNA

Lentivirus stocks were prepared following the manufacturer’s protocol, as described previously [27]. Briefly, 1.5×10^6^ 293T cells were plated in 10-cm dishes. 24 hours later, cells were co-transfected with shRNA constructs (3 μg) together with pCMV-dR8.2 dvpr (3 μg) and pCMV-VSV-G (0.3 μg) helper constructs using Lipofectamine 2000 reagent. Two days later, viral stocks were harvested from the culture medium, which was filtered to remove non-adherent 293T cells. To select NSCLC cells that stably expressed shRNA constructs, cells were plated at subconfluent densities and infected with a cocktail of 1 ml of virus-containing medium, 3 ml of regular medium, and 8 μg/ml polybrene. Selection with 2 μg/ml of puromycin was started 48 hours after lentivirus infection. After about 4 weeks of selection, monolayers of stably infected pooled clones were harvested for use and cryopreserved. Western blot was used to evaluate LKB1 stable knockdown in NSCLC cells.

### GFP-LKB1 adenovirus

The GFP-LKB1 adenovirus was constructed as previously described [24]. Briefly, GFP-LKB1 plasmid was digested with Kpn1, and the 5’-overhang was filled in with Klenow DNA polymerase. The GFP-LKB1 fragment was released from the vector backbone by NheI digestion, and cloned into pShuttle vector digested with XbaI and EcoRV. The pShuttle-GFP-LKB1 plasmid was transformed into BJ5183-AD-1 cells (Stratagene, cat# 200157) to generate AdEasy-GFP-LKB1 plasmid. This plasmid was transfected into 293 cells to generate adenovirus containing GFP-LKB1.

### Transient transfection

Transfections were carried out with Lipofectamine 2000 reagent according to the manufacturer's instructions (Invitrogen). Briefly, cells grown to 90% confluence in 6-well plates were transfected with 1.0 μg/well plasmids with 3 μl Lipofectamine 2000 and replaced with fresh growth medium after 4–6 hours.

### Small interfering RNA treatment

SiRNA specific for AMPK-α1 (PRKAA1) and AMPK-α2 (PRKAA2) were purchased from Applied Biosystem (AB), with the corresponding sequences: 5’GAGUCUACAGUUAUACCAAtt-3’ and 5’-GCAUAUGGUUGUUCAUCGAtt-3’. Lamin A/C siControl (Santa Cruz Biotechnology) was used to control for any nonspecific off-target effects of siRNA transfection. Cells were transiently transfected with chemically synthesized AMPK siRNAs (200 pmol) or with the nonsilencing control siRNA using Lipofectamine 2000 reagent (Invitrogen) according to the manufacturer’s instructions. Specifically, H522, H520, Calu-1 and H1650 cells were grown to 60–70% confluence in 6-well plates. Lipofectamine 2000 reagent was incubated with Opti-MEM1 for 5 min, and a mixture of siRNA was then added. After incubation for 20 min at room temperature, the mixture was diluted with medium and added to each well.

### Western blot

Proteins were resolved by polyacrylamide gel electrophoresis and transferred onto polyvinylidene difluoride (PVDF) membranes (Millipore, Germany). The membranes were blocked in Tris-buffered saline containing 0.2% Tween 20 and 5% fat-free dry milk and incubated first with primary antibodies and then with horseradish peroxidase-conjugated secondary antibodies. Specific proteins were visualized with enhanced chemiluminescence detection reagent according to the manufacturer’s instructions (Pierce Biotechnology, USA).

## Results

### 2-DG inhibits ERK activation in LKB1 wild-type NSCLC cells but not in LKB1 mutant cells

**Fig 1:**
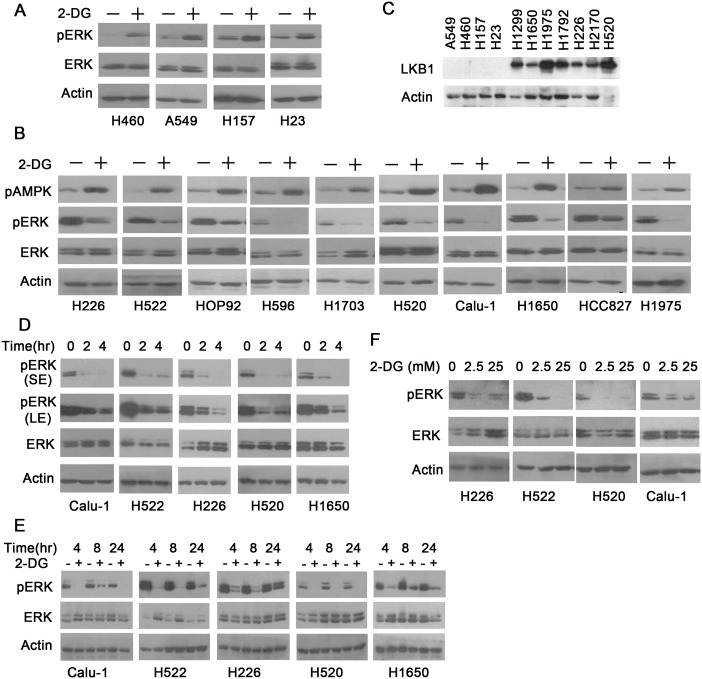
2-DG inhibits ERK activation in LKB1 wild-type NSCLC cells but not in LKB1 mutant cells. A. H460, A549, H157 and H23 cells were treated with 25 mM 2-DG for 2 hours, and p-ERK (Thr202/Tyr204) was examined by Western blot. B. H226, H522, HOP92, H596, H1703, H520, Calu-1, H1650, HCC827 and H1975 cells were treated with 25 mM 2-DG for 2 hours, and p-ERK 1/2 and p-AMPK (Thr172) were examined by Western blot. C. A549, H460, H157, H23, H1299, H1650, H1975, H1792, H226, H2170 and H520 cells were harvested and lysed, and LKB1 protein expression was examined by Western blot. D. Calu-1, H522, H226, H520 and H1650 cells were treated with 25 mM 2-DG for the indicated length of time (0 hours, 2 hours, 4 hours), and p-ERK was examined by Western blot. E. Calu-1, H522, H226, H520 and H1650 cells were treated with 25 mM 2-DG for the indicated length of time (4 hours, 8 hours, 24 hours), and p-ERK was examined by Western blot. F. H226, H522, H520 and Calu-1 were treated with 2-DG at the indicated concentrations (0 mM, 2.5 mM, 25 mM) for 2 hours, and p-ERK was examined by Western blot.

We previously demonstrated that 2-DG activates IGF1R signaling [17], thus we expected that 2-DG treatment should uniformly promote the activation of downstream MEK-ERK signaling. To investigate the effect of 2-DG on ERK phosphorylation in NSCLC cell lines of diverse genetic background (Table 1), a panel of cell lines was treated with 25 mM 2-DG for 2 hours, and the phosphorylation of ERK1/2 at Thr202/Tyr204 was examined with Western blot. Consistent with our previous findings, 2-DG treatment indeed led to increases in ERK phosphorylation in H460, A549, H157 and H23 cells (Fig 1A). However, in H226, H522, HOP92, H596, H1703, H520, Calu-1, H1650, HCC827 and H1975 cells, 2-DG treatment resulted in decreases in ERK phosphorylation (Fig 1B). Notably, H460, A549, H157 and H23 cells contain bi-allelic inactivation of LKB1, which abolished the expression of LKB1 protein (Fig 1C). Thus, these results suggest that the differential effect of 2-DG on various cancer cells was not related to p53 or EGFR mutation status but correlated with LKB1 status. We used the phosphorylation of AMPK at Thr172 as a surrogate marker for LKB1 function [24]. AMPK is not phosphorylated in LKB1-mutant NSCLC cells after the addition of 2-DG [16]. As shown in Fig 1B, 2-DG treatment led to a significant increase in AMPK phosphorylation in LKB1-WT cells, indicating that LKB1 is functional in these cells. These results suggest that 2-DG treatment is capable of inhibiting ERK activation via a functional LKB1 protein.

**Table 1:** Genetic alterations in NSCLC cell lines.

Cell Line	*LKB1*	*TP53*	*KRAS*	*HRAS*	*NRAS*	*EGFR*
**H226**	WT	WT	WT	WT	WT	WT
**H522**	WT	fs	WT	WT	WT	WT
**HOP92**	WT	R175L	WT	WT	WT	WT
**H596**	WT	G245C	WT	WT	WT	WT
**H1703**	WT	fs	WT	WT	WT	WT
**H520**	WT	W146*	WT	WT	WT	WT
**Calu-1**	WT	HD	G12C	WT	WT	WT
**H1650**	WT	fs	WT	WT	WT	E746-A750-del
**HCC827**	WT	V218-del	WT	WT	WT	E746-A750-del
**H1975**	WT	R273H	WT	WT	WT	L858R
**A549**	Q37*	WT	G12S	WT	WT	WT
**H460**	Q37*	WT	Q61H	WT	WT	WT
**H157**	del[24]	E298*	G12R	WT	WT	WT
**H23**	W332*	M246I	G12C	WT	WT	WT

Data source: Catalogue of somatic mutations in cancer: fs: frame shift;

*: nonsense mutation;

HD: homozygous deletion; del: deletion

To characterize the inhibitory effect of 2-DG on ERK phosphorylation in LKB1-WT cells, Calu-1, H522, H226, H520 and H1650 cells were treated with 2-DG (25 mM) for varying lengths of time. As shown in Fig 1D, ERK phosphorylation was moderately suppressed upon 2-DG treatment for 2 hours, and was significantly inhibited after 4-hour treatment. In addition, this p-ERK inhibition continued to be detectable even 8–24 hours after 2-DG treatment (Fig 1E). We also treated LKB1-WT cells (H226, H522, H520 and Calu-1) with 2-DG at the indicated concentrations for 2 hours. As shown in Fig 1F, 2.5 mM 2-DG was sufficient to suppress ERK phosphorylation in all four cell lines, but 25 mM 2-DG treatment led to a more complete suppression in H520 cells. These results indicate that 2-DG inhibits ERK phosphorylation in a time and dose-dependent manner in LKB1-WT cells.

### 2-DG—induced ERK inhibition is independent of PI3K/AKT activation

**Fig 2:**
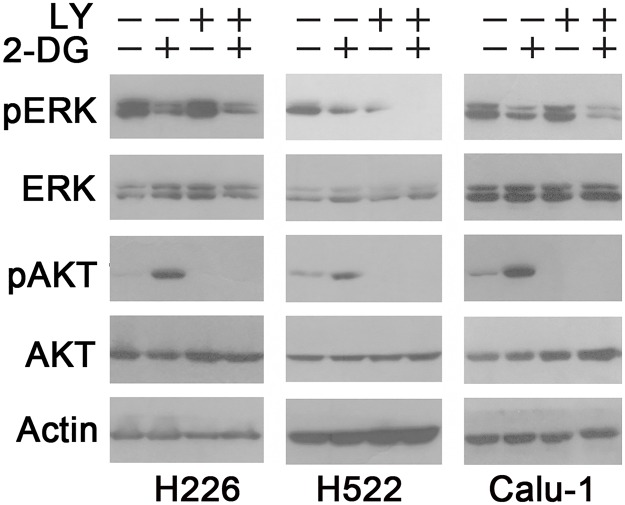
2-DG—induced ERK inhibition is independent of PI3K/AKT signaling. H226, H522 and Calu-1 cells were pretreated with or without LY294002 (10 μM) for 30 minutes before 2-DG treatment. p-AKT and p-ERK levels were examined by Western blot.

We previously demonstrated that 2-DG induces AKT phosphorylation in both LKB1 mutant and LKB1-WT NSCLC cells [16]. AKT has been reported to negatively regulate MEK/ERK signaling through phosphorylating RAF [28]. To investigate the role of AKT in 2-DG—mediated ERK inhibition, LKB1-WT NSCLC cells (H226, H522 and Calu-1) were pretreated with or without LY294002(LY), a small molecular inhibitor of PI3K, for 30 minutes before 2-DG treatment. As shown in Fig 2, 2-DG treatment indeed led to increased phosphorylation of AKT. In the presence of LY294002, the basal level of AKT phosphorylation was abolished. LY294002 pretreatment did not alter 2-DG—induced ERK inhibition, suggesting that this inhibition is not mediated by PI3K/AKT signaling.

### LKB1 negatively regulates ERK phosphorylation

**Fig 3:**
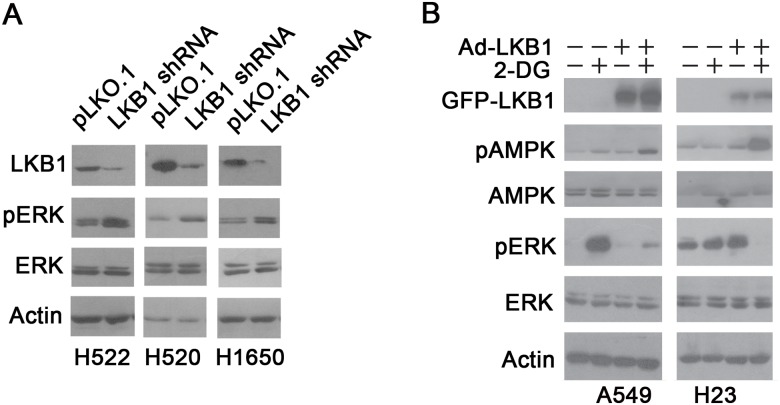
LKB1 negatively regulates ERK phosphorylation. A. Isogenic LKB1 stable knockdown NSCLC cell lines (H522-pLKO.1 and H522-LKB1 shRNA, H520- pLKO.1 and H520-LKB1 shRNA, H1650-pLKO.1 and H1650-LKB1 shRNA) were established using a lentivirus system. LKB1 protein expression and p-ERK levels were examined with Western blot. B.A549 and H23 cells were infected with adenovirus expressing GFP-LKB1 fusion protein or control adenovirus containing only GFP, and treated with 25 mM 2-DG for 2 hours. LKB1, p-AMPK and p-ERK levels were examined by Western blot.

The finding that 2-DG requires functional LKB1 to inhibit ERK in NSCLC cells (Fig 1) prompted us to investigate whether LKB1 is involved in negative regulation of ERK phosphorylation. To test this hypothesis, we established isogenic LKB1 stable knockdown NSCLC cells (H522 pLKO.1 and H522 LKB1 shRNA, H520 pLKO.1 and H520 LKB1 shRNA, H1650 pLKO.1 and H1650 LKB1 shRNA) using a lentivirus system. As shown in Fig 3A, compared with the control cells, LKB1 protein levels were substantially reduced in cells stably expressing LKB1 shRNA. LKB1 downregulation correlated with an increase in the basal level of p-ERK.

Next, we sought to evaluate the impact of ectopic LKB1 expression on 2-DG—induced ERK activation in LKB1 null NSCLC cells. A549 and H23 cells were infected with adenovirus expressing GFP-LKB1 fusion protein or control adenovirus containing only GFP [24], and the cells were then treated with 25 mM 2-DG for 2 hours. As shown in Fig 3B, compared with the control group, ectopic expression of LKB1 significantly attenuated 2-DG—induced ERK activation in both LKB1 mutant cell lines. Together, these results indicate that LKB1 negatively regulates ERK phosphorylation.

### AMPK acts downstream of LKB1 to inhibit ERK phosphorylation

**Fig 4:**
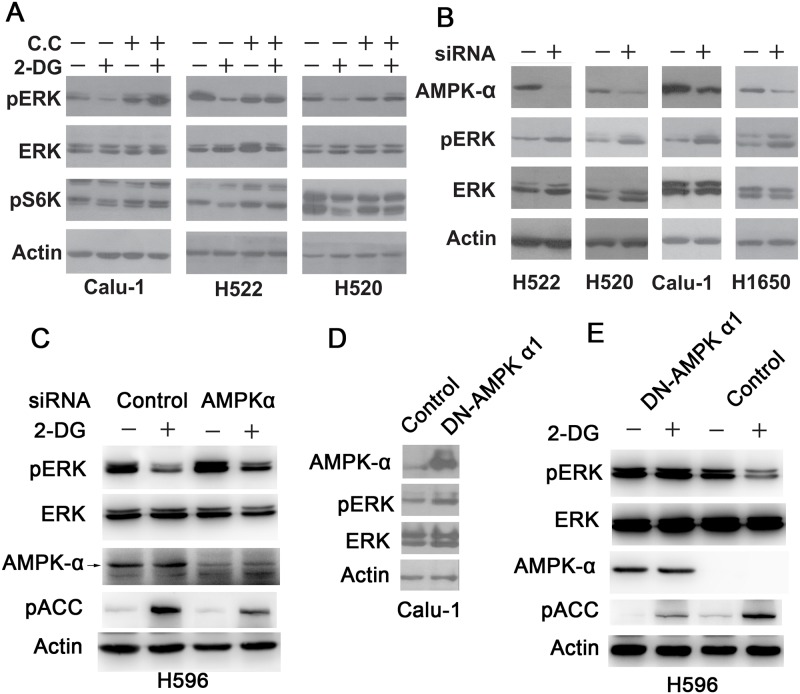
AMPK acts downstream of LKB1 to inhibit p-ERK. A. H522, Calu-1 and H520 cells were pretreated with or without 10 μM Compound C (C.C) for 30 minutes before 2-DG treatment. P-S6K and p-ERK levels were examined by Western blot. B. H522, H520, Calu-1 and H1650 cells were transiently transfected with control siRNA or AMPKα siRNA. AMPK protein expression and p-ERK levels were examined by Western blot. C. H596 cells were transiently transfected with control siRNA or AMPKα siRNA, and treated with 25 mM 2-DG for 2 hours. AMPK protein expression and p-ERK levels were examined by Western blot. D. Calu-1 cells were transiently transfected with plasmid encoding control vector or dominant negative AMPKα 1(DN-AMPK), and AMPKα protein expression and p-ERK levels were examined by Western blot. E. H596 cells were transiently transfected with plasmid encoding control vector or dominant negative AMPK α 1 (DN-AMPK), and treated with 25 mM 2-DG for 2 hours. AMPKα protein expression and p-ERK levels were examined by Western blot.

AMPK, one of the canonical substrates of LKB1 as described above, has previously been shown to be potentially involved in regulating ERK signaling [29–31]. To examine the role of AMPK in 2-DG—induced ERK inhibition, Compound C (C.C), a small molecule inhibitor of AMPK, was used. LKB1-WT Calu-1, H522 and H520 cells were pretreated with or without C.C (10 μM) for 30 minutes before 2-DG treatment. This pharmacological inhibition of AMPK activity greatly diminished the inhibitory effect of 2-DG on ERK, indicating that ERK inhibition by 2-DG is probably mediated by AMPK (Fig 4A).

To further evaluate the role of AMPK in regulating ERK phosphorylation, LKB1 WT cells were transiently transfected with control siRNA or AMPKα siRNA, in the absence or presence of 2-DG. As shown in Fig 4B, transient depletion of AMPKα by siRNA resulted in enhanced basal levels of ERK phosphorylation. Furthermore, suppression of AMPK expression and activity (as demonstrated by the phosphorylation of its key substrate ACC) by RNAi partially attenuated ERK inhibition by 2-DG (Fig 4C).

LKB1 WT cells were also transiently transfected with plasmids encoding control vector or dominant negative N157A AMPKα 1 (DN-AMPK). As shown in Fig 4D, compared with control, the expression of DN-AMPK promoted ERK phosphorylation significantly in Calu-1 cells. Consistently, 2-DG—mediated ERK inhibition was abrogated by DN-AMPK (Fig 4E). Taken together, these data indicate that AMPK acts downstream of LKB1 to negatively regulate ERK activation.

### IGF-1–induced ERK activation is inhibited by 2-DG

**Fig 5:**
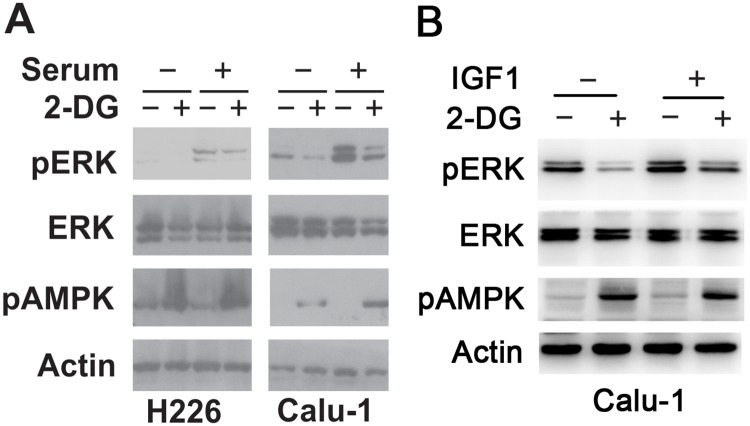
IGF-1–induced ERK activation is inhibited by 2-DG. A. H226 and Calu-1 cells were serum-starved for 4 hours and then pretreated with or without 2-DG before addition of fetal bovine serum (FBS). P-AMPK (Thr172) and p-ERK levels were examined by Western blot. B. Calu-1 cells were serum-starved for 4 hours, pretreated with or without 2-DG, and then challenged with 20 ng/ml IGF-1 for 10 min. P-ERK levels were examined by Western blot.

To investigate the effect of 2-DG on ERK activation induced by growth factors, LKB1 WT cells (H226 and Calu-1) were serum-starved for 4 hours and then pretreated with or without 2-DG before the addition of fetal bovine serum (FBS). As shown in Fig 5A, in the absence of 2-DG, FBS potently stimulated ERK activation. 2-DG pretreatment induced AMPK activation as assessed by the increased level of p-AMPK, and greatly attenuated serum-dependent ERK activation. Next, we sought to evaluate the impact of 2-DG on ERK activation induced by IGF1 (20 ng/ml). As shown in Fig 5B, in the absence of 2-DG, IGF-1 increased ERK phosphorylation. 2-DG pretreatment significantly blocked IGF-1–dependent ERK phosphorylation. These results suggest that 2-DG is capable of inhibiting IGF-1–dependent ERK signaling.

### A subset of K-Ras oncogenic mutations blocks 2-DG—induced LKB1/AMPK signaling

**Fig 6:**
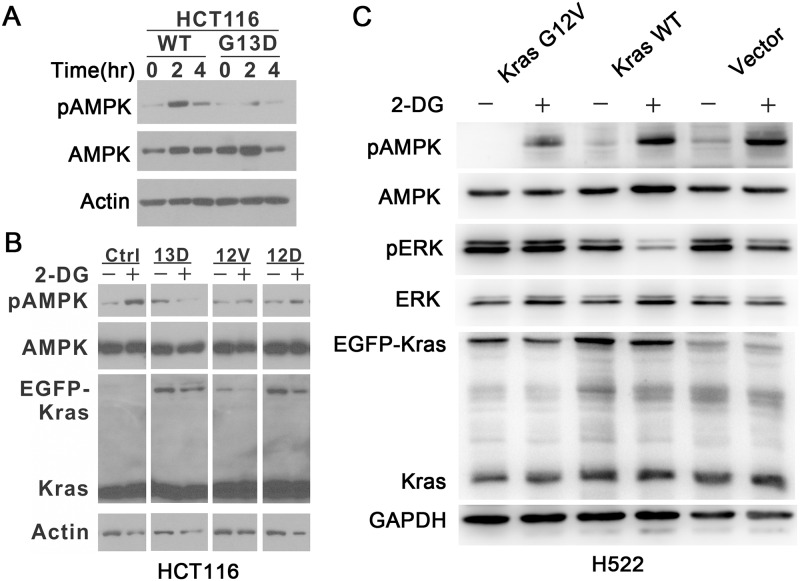
A subset of oncogenic K-Ras mutations blocks 2-DG—induced LKB1/AMPK signaling. A. Isogenic K-Ras WT and K-Ras-G13D HCT116 colon carcinoma cells were treated with 25 mM 2-DG for the indicated length of time (0 hours, 2 hours, 4 hours), and p-AMPK level was examined by Western blot. B. K-Ras WT HCT116 cells were transfected with plasmids encoding vector or the indicated EGFP K-Ras mutant (13D, 12V or 12D), and then treated with 25 mM 2-DG for 2 hours. Ras protein expression and p-AMPK levels were examined by Western blot. C. H522 cells were transfected with plasmids encoding Kras G12V, WT Kras or vector, and then treated with 2-DG. Kras, p-ERK and p-AMPK levels were examined by Western blot.

We previously demonstrated that 2-DG induced ERK phosphorylation in three LKB1-WT cell lines, H1299, H1792 and H358 [17]. A close examination of these three cell lines indicated that all of them contain either N-Ras or K-Ras mutations. In contrast, among the 10 cell lines evaluated in Fig 1B, 9 contain WT K-Ras (Table 1). This finding led us to evaluate the effect of K-Ras mutation on 2-DG—induced LKB1/AMPK activation. Isogenic K-Ras WT and K-Ras-G13D HCT116 colon carcinoma cells [32] were treated with 25 mM 2-DG for the indicated lengths of time, and p-AMPK levels were examined with Western blot. As shown in Fig 6A, in K-Ras WT HCT116 cells, 2-DG induced AMPK phosphorylation robustly. In contrast, in K-Ras mutant cells, the p-AMPK level was only slightly increased by 2-DG. This result suggests that K-Ras G13D mutant is capable of attenuating 2-DG—induced AMPK activation.

To further evaluate the impact of various K-Ras mutations on 2-DG—induced LKB1/AMPK activation, HCT116 cells with only WT endogenous K-Ras were transfected with different cancer-derived K-Ras mutants, including 13D, 12V and 12D, and then were treated with 25 mM 2-DG for 2 hours. As shown in Fig 6B, compared to the control group, overexpression of G13D and G12V significantly attenuated 2-DG—induced AMPK phosphorylation. In contrast, the G12D mutant had a limited effect on 2-DG—induced AMPK phosphorylation.

To confirm our findings in NSCLC cells, LKB1 WT/Kras WT H522 cells were transfected with plasmids encoding Kras G12V, WT Kras or vector, and then treated with 2-DG. As shown in Fig 6C, in cells transfected with WT Kras or vector, 2-DG robustly induced AMPK activation and significantly inhibited p-ERK. In contrast, in cells with over-expression of Kras G12V, 2-DG treatment neither significantly increased AMPK phosphorylation nor suppressed ERK phosphorylation. These results demonstrate that a subset of K-Ras mutants is capable of inhibiting 2-DG—induced AMPK phosphorylation.

## Discussion

Glucose is the major source of energy for cancer cells. Warburg demonstrated that cancer cells largely depend on aerobic glycolysis to generate ATP [2]. A direct consequence of this discovery is the development of therapeutic strategies that target glycolysis. 2-DG, the best known glycolysis inhibitor, has been shown to interfere with anabolic processes, disrupt antioxidant defenses and induce energy stress by blocking glycolysis [13]. However, 2-DG also has unanticipated side effects that remain to be deciphered. Herein, we report that 2-DG inhibits the ERK cascade in a subset of NSCLC cancer cells with wild-type LKB1 and K-Ras.

One intriguing finding of our study is the potential cross-talk between LKB1/AMPK and ERK signaling. LKB1 is inactivated in 20–30% of NSCLC, ranking it as the third most frequently mutated gene in lung adenocarcinoma after p53 and K-Ras [22]. As a tumor suppressor, LKB1 plays significant roles in inhibiting lung cancer initiation and metastasis [33]. However, the underlying molecular mechanisms remain to be fully elucidated. Herein, we demonstrate that LKB1 negatively regulates ERK activation in NSCLC cells. Given the important role of the ERK signaling pathway in the regulation of cancer cell proliferation, survival and metastasis [21], our findings suggest that the downregulation of ERK signaling may contribute to the multiple biological functions of LKB1 in lung cancer cells. For example, sustained ERK activation in cancer cells has been shown to enhance the induction of MMPs in the surrounding environment, leading to degradation of the extracellular matrix (ECM) [34]. Therefore, our findings suggest that ERK signaling might be an important mediator of lung cancer progression and metastasis provoked by LKB1 deficiency, offering novel molecular insights into the tumor suppressive role of LKB1.

Mechanistically, our data indicate that LKB1 mediates this effect through the energy sensor AMPK [35]. We provide evidence that blocking AMPK function with AMPK inhibitor, AMPK siRNA or DN-AMPK α1 plasmid results in increased ERK phosphorylation. ERK downregulation by LKB1/AMPK could represent the mechanism by which cell proliferation is inhibited when cancer cells are exposed to stresses causing ATP depletion. The cross-talk between AMPK and ERK has been explored by several groups, but the findings are contradictory regarding how the two signaling pathways interact. Consistent with our findings, a recent study [31] reported that AMPK attenuates ERK signaling in MEF cells by phosphorylating BRAF Ser729. Paradoxically, prolonged treatment with AICAR and metformin, two other AMPK activators, has been shown to enhance ERK phosphorylation in melanoma cells by inducing degradation of dual-specificity phosphatase (DUSP) 6 [36]. Consequently, the regulation of ERK signaling by LKB1/AMPK is highly complex and further studies are needed to dissect the precise mechanisms that link the two signaling pathways.

Another intriguing finding of this study is that a subset of Ras mutations is capable of overriding 2-DG—induced LKB1/AMPK activation. Thus, it is conceivable that there is an interactive regulation between LKB1/AMPK and RAS-RAF-MEK-ERK signaling in cancer cells. Intriguingly, LKB1 loss is frequently accompanied by K-Ras mutations in NSCLC. In addition, concomitant loss of LKB1 significantly accelerates oncogenic K-Ras G12D-driven lung cancer progression in a mouse model [33]. Moreover, LKB1 loss detected by immunohistochemistry is a biomarker for more aggressive biology in K-Ras-mutant lung adenocarcinoma [37]. These reports suggest a potential link between LKB1 and K-Ras in lung cancer. In our study, we provide novel evidence that a subset of oncogenic K-Ras mutants (G13D and G12V) can attenuate LKB1/AMPK signaling in colon carcinoma cells, whereas G12D cannot. The mechanism underlying the differential effect of distinct oncogenic RAS mutations on LKB1/AMPK signaling remains unknown. K-Ras G12V mutations have been associated with a worse prognosis than G12D mutations in lung cancer [38]. It thus seems that particular amino acid substitutions might dictate specific oncogenic outcomes [39]. In agreement with our study, ERK has been previously reported to inactivate LKB1/AMPK signaling in melanoma cells by phosphorylating LKB1 and compromising the ability of LKB1 to bind and activate AMPK activation [40]. In contrast, a recent finding demonstrates that oncogenic H-Ras induces LKB1 polyubiquitination and activation by activating Skp2-SCF under energy stress [41]. Therefore, the regulation of LKB1 by hyperactive Ras is intricate and complicated. It is hypothesized that, on one hand, Ras-driven cancer cells must inactivate the tumor suppressor LKB1 to grow; on the other hand, they need to activate LKB1/AMPK signaling to counteract metabolic stress-induced cell death.

Overall, 2-DG exerts inhibitory effects on the growth or survival of all lung cancer cells tested(as shown in S1 Fig), but the molecular basis of 2-DG mediated growth suppression is a complex topic that cannot be stratified by LKB1-mutation status alone. For example, we initially hypothesized that LKB1-null cells would be more susceptible to 2-DG—mediated suppression because these cells lack an LKB1/AMPK-mediated metabolic checkpoint to promote cell survival under those circumstances. Our published study later refuted that hypothesis by demonstrating that 2-DG is also capable of activating various prosurvival pathways in LKB1-mutant cells through the activation of IGF1R [16,17].

The situation in LKB1-wild type cells turns out to be equally complex. Previous studies predicted that the activation of LKB1-AMPK signaling will suppress mTOR signaling and inhibit cell proliferation [42–44]. In this study, we discovered that the activation of AMPK signaling by 2-DG through LKB1 also suppresses MEK/ERK signaling, and this suppression may also contribute to the growth suppression in LKB1-wild type cells. However, another important finding of this study is that a subset of Kras mutants is capable of suppressing the function of wild-type LKB1 to maintain the aberrant activation of MEK/ERK signaling. In summary, these findings highlight the fact that lung cancers have heterogeneous genetic alterations, and LKB1 mutational status alone cannot be used to predict the response of these cell lines to 2-DG treatment.

In summary, our study shows that the glycolysis inhibitor 2-DG suppresses ERK phosphorylation in a subset of NSCLC cells with wild-type LKB1 and K-Ras. The study reveals the potential cross-talk between LKB1/AMPK and ERK signaling, and offers novel insights into the tumor suppressor role of LKB1. In addition, these findings help to better define the mechanism of the biological effects of 2-DG and provide a rationale for lung cancer targeted therapy.

## Supporting Information

S1 Fig The effect of 2-DG on the survival of various LKB1 wild-type or LKB1 mutant NSCLC cells. (JPG)
