# Supplementary figures and images for: 2-Deoxyglucose Suppresses ERK Phosphorylation in LKB1 and Ras Wild-Type Non-Small Cell Lung Cancer Cells
Source: PLoS One. 2016 Dec 29;11(12):e0168793. doi: 10.1371/journal.pone.0168793 (PMC5198974; doi:10.1371/journal.pone.0168793)

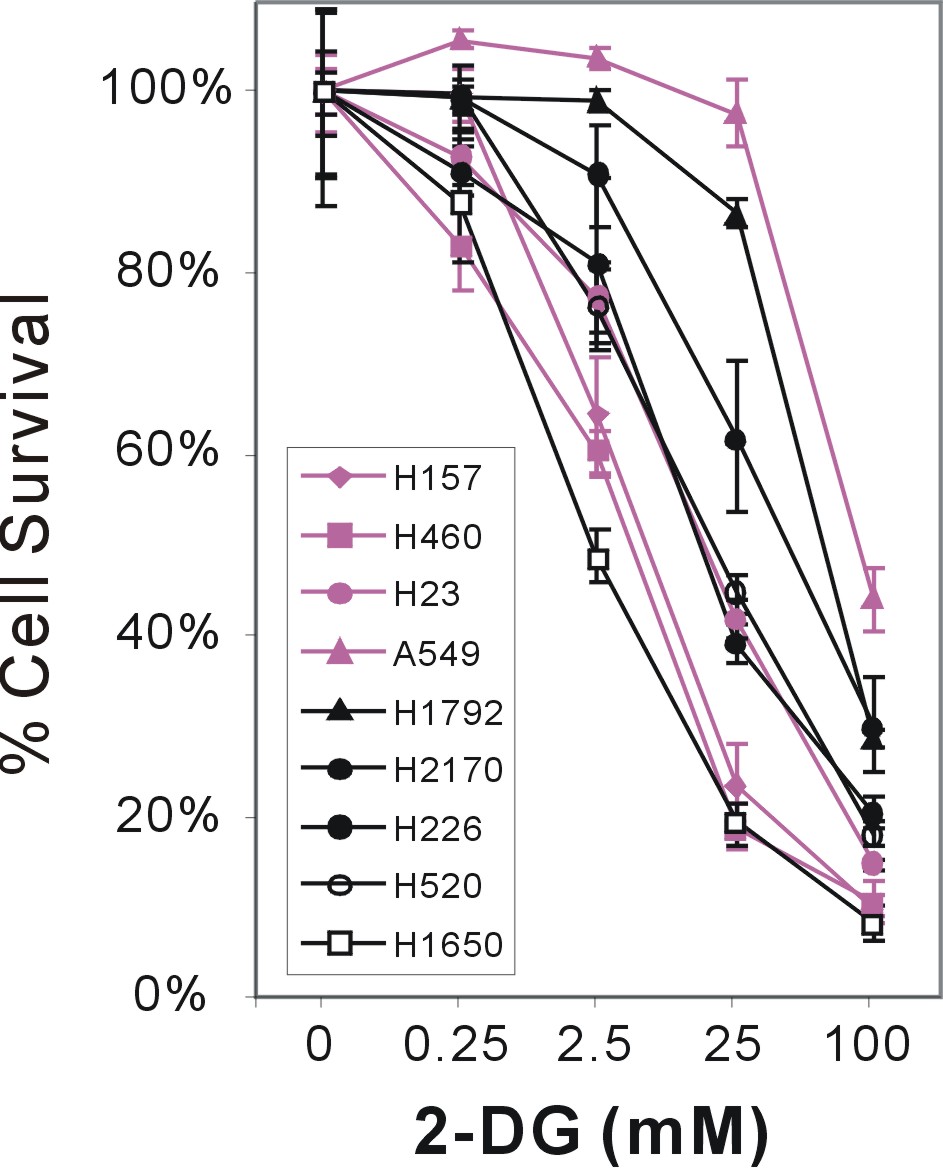

Supplement: S1 Fig — (JPG) [file pone.0168793.s001.jpg]
